# Influence of exercise training on nitric oxide pathways and their physiological effects

**DOI:** 10.1016/j.redox.2026.104041

**Published:** 2026-01-23

**Authors:** Jonas Benjamim, Stephen J. Bailey, Leonardo da Silva Gonçalves, Mia Burleigh, Mario Siervo, Andrew R. Coggan, Raúl Bescos

**Affiliations:** a Institute for Physical Activity and Nutrition (IPAN), Deakin University, Geelong, Victoria, Australia; b School of Sport, Exercise and Health Sciences, Loughborough University, Loughborough, United Kingdom; c Department of Internal Medicine, Ribeirao Preto Medical School, University of São Paulo, SP, Brazil; d Sport and Physical Activity Research Institute, University of the West of Scotland, Blantyre, Scotland, United Kingdom; e School of Population Health, Curtin University, Perth, Western Australia, Australia; f Indiana Center for Musculoskeletal Health, Indiana University School of Medicine, Indianapolis, IN, United States; g School of Health Professions, Faculty of Health, University of Plymouth, Plymouth, PL4 6AB, United Kingdom

**Keywords:** Physical exercise, Nitrogen monoxide, Redox biology, Microbiota, Inorganic nitrate

## Abstract

Nitric oxide (NO) is a critical signalling molecule in cardiovascular, metabolic, and muscular function. Endogenous NO production occurs via two primary metabolic pathways: 1) the classical nitric oxide synthases (NOS) pathway, and 2) the alternative (nitrate–nitrite–NO) pathway, in which inorganic nitrate (NO_3_^−^) is sequentially reduced to nitrite (NO_2_^−^) and other NO intermediates (e.g., S-nitrosothiol). The latter pathway relies heavily on the oral microbiota, which catalyze the two-electron partial reduction of NO_3_^−^ to NO_2_^−^, which is influenced by oral physiology, microbial composition and salivary flow. While the role of exercise training in enhancing NOS-derived NO is well established, emerging evidence suggests that it may also augment NO bioavailability through the NO_3_^−^–NO_2_^-^–NO pathway. Furthermore, exercise training may influence the composition and functionality of oral microbiota, thereby indirectly modulating NO metabolism and oral health. However, the synergistic effects of exercise and oral microbiota on NO production remain underexplored. This review synthesises current evidence on how physical exercise may modulate both NO pathways and discusses the broader physiological implications.

## Highlights

• The integrative effects of exercise on nitric oxide-producing pathways remain underexplored.
• This review provides a comprehensive overview of how exercise enhances nitric oxide homeostasis.
• Exercise training stimulates NO production through both nitric oxide synthase–dependent and nitrate–nitrite–nitric oxide pathways.
• Exercise and dietary nitrate may exert synergistic effects on nitric oxide signalling.
• The review discusses the broader physiological implications of these integrated mechanisms.

## Graphical abstract

**Figure:**
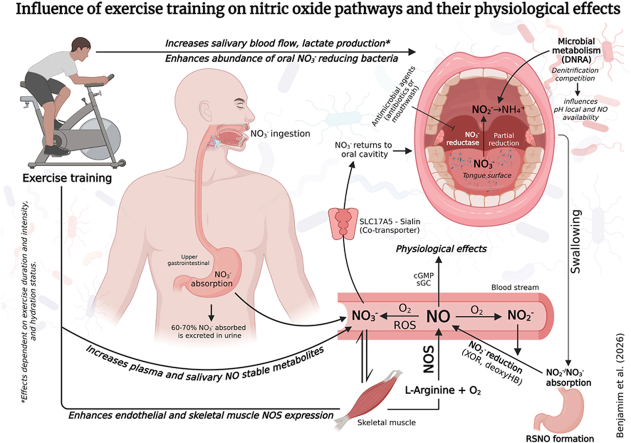

## Abbreviations:

ATP: Adenosine triphosphate
BRJ: Beetroot juice
BH_2_: Dihydrobiopterin
BH_4_: Tetrahydrobiopterin (NOS cofactor)
cGMP: Cyclic guanosine monophosphate
DeoxyHb: Deoxygenated hemoglobin
DeoxyMb: Deoxygenated myoglobin
DNRA: Dissimilatory nitrate reduction to ammonium
eNOS: Endothelial nitric oxide synthase
FMD: Flow-mediated dilation
Hb: Hemoglobin
HFrEF: Heart failure with reduced ejection fraction
HIIT: High-intensity interval training
HNO: Nitroxyl
HNO_2_: Nitrous acid
iNOS: Inducible nitric oxide synthase
Mb: Myoglobin
mtNOS: Mitochondrial nitric oxide synthase
N_2_O_3_: Dinitrogen trioxide
NADPH: Nicotinamide adenine dinucleotide phosphate (reduced form)
NH_4_^+^: ammonium
NiR: Nitrite reductase
NO: Nitric oxide
NO_2_^−^: Nitrite
NO_3_^−^: Nitrate
NO^+^: Nitrosonium ion
NOS: Nitric oxide synthase
NR: Nitrate reductase (bacterial)
NRB: Nitrate-reducing bacteria
ONOO^−^: Peroxynitrite
pH: Hydrogen ion concentration
RBC: Red blood cells
ROS: Reactive oxygen species
RSNO: S-nitrosothiols
sGC: Soluble guanylyl cyclase
SIT: Sprint interval training
$\dot{V}$O_2_: Oxygen uptake
$\dot{V}$O_2_max: Maximal oxygen uptake
$\dot{V}$O_2_peak: Peak oxygen uptake
XO: Xanthine oxidase
XOR: Xanthine oxidoreductase

## 1 Introduction

Nitric oxide (NO) is a highly diffusible gaseous signalling molecule that plays pivotal roles in cardiovascular homeostasis, neurotransmission, muscle contractile function, immune response modulation, metabolic regulation and various other physiological processes [1]. Endogenously, NO is synthesised through two distinct physiological pathways. The first is the l-arginine-NOS pathway, in which l-arginine is oxidised to l-citrulline and NO by nitric oxide synthase (NOS) enzymes. This enzymatic reaction requires molecular oxygen and multiple cofactors such as tetrahydrobiopterin (BH_4_), flavin adenine dinucleotide (FAD), flavin mononucleotide (FMN), and nicotinamide adenine dinucleotide phosphate (reduced form) (NADPH) [2]. In addition, a second and oxygen-independent pathway has been elucidated. In this alternative pathway, inorganic nitrate (NO_3_^−^) from endogenous (NO synthesis) or exogenous sources (diet) is partially reduced to nitrite (NO_2_^−^), carried out primally by facultative anaerobic bacteria on the tongue surface. Species from the genera *Rothia* and *Neisseria* have shown higher NO_3_^−^-reducing activity [3]. Once NO_2_^−^ is formed in the oral cavity, it can be swallowed with saliva and further reduced to NO in the acidic environment of the stomach or under low oxygen tension in peripheral tissues [[4], [5], [6]]. During high-intensity exercise, intramuscular oxygen tension (PO_2_) can fall to ∼2 % which may reflect physiological normoxia rather than true hypoxia. In such conditions, plasma NO_2_^−^ tends to decline after NO_3_^−^ supplementation, consistent with a PO_2_-dependent role of NO_2_^−^ in modulating muscle function [7].

Intramuscular NO production contributes to the regulation of numerous physiological processes, such as local blood flow regulation, glucose uptake and mitochondrial biogenesis, which can conflate to enhancing muscle function and exercise capacity [[8], [9], [10]]. Human and animal studies have shown higher concentrations of NO_3_^−^ in skeletal muscles compared to blood and a number of other tissues, suggesting that skeletal muscle may serve as a reservoir for nitrogen oxides that can be mobilised for NO production under specific physiological conditions [2,11,12]. The oral microbiota, through its critical role in reducing NO_3_^−^ to NO_2_^−^, represents a key determinant of NO_3_^−^ bioactivation in humans. Major interspecies differences limit translation of preclinical animal model mechanisms to humans. Unlike rodents, humans actively concentrate NO_3_^−^ in saliva—10 to 20 times higher than plasma—allowing oral bacterial partial reduction to NO_2_^−^ [13]. Although limited, evidence has shown that in rodents, both skeletal muscle NO_3_^−^ and NO_2_^−^levels may increase following NO_3_^−^ supplementation [14]. On the other hand, in human skeletal muscle, there are elevations only in NO_3_^−^ [15,16]. This difference implies that humans may rely more on oral NO_3_^−^ reduction to drive NO synthesis from elevated NO_3_^−^ sources (whether endogenous or exogenous). In contrast, rodents seem to exhibit a greater intrinsic capacity for NO_2_^−^ generation within skeletal muscle [17]. In this regard, skeletal muscle from nNOS knockout (nNOS^−/−^) mice exhibits significantly lower NO_3_^−^ levels than wild-type controls [18]. Consistent with these interspecies differences, the presence and functional relevance of xanthine oxidoreductase (XOR) in human skeletal muscle remain poorly characterised, further limiting direct extrapolation of rodent nitrite-reduction mechanisms to humans. These findings suggest that skeletal muscle may play a dual role as both a site of NO production and a reservoir for NO precursors. This dual function could be particularly relevant during periods of increased metabolic demand, such as exercise.

Currently, it is well established that exercise training enhances NO production by upregulating NOS activity [19]. In addition, emerging evidence also suggests that exercise training may augment NO synthesis through the NO_3_^−^–NO_2_^-^–NO pathway. However, the integrated effects of exercise on both NO-producing pathways remain underexplored. As such, this review aims to provide an integrated overview of how exercise may enhance NO homeostasis via both the NOS and NO_3_^−^-NO_2_^-^-NO pathways, and to discuss the broader physiological implications of these interactions. The review has been organised to provide a coherent progression from fundamental mechanisms to applied physiological outcomes. The first two sections describe the principles of NO homeostasis, emphasising human physiology while briefly referencing animal studies only when necessary to illustrate key interspecies differences. The third section explores how exercise training modulates the composition and function of the oral microbiota, highlighting its influence on NO_3_^−^ reduction and systemic NO homeostasis. Finally, the last two sections synthesise evidence on the independent and combined stimulation of the NO_3_^−^–NO_2_^-^–NO and NOS–dependent pathways, demonstrating how these mechanisms converge to enhance exercise performance and support cardiovascular and metabolic health.

## 2 Effect of exercise training on the l-arginine/NOS pathway

The l-arginine/NOS pathway was identified in the 1980s [20]. In this pathway, the amino acid l-arginine is oxidised to NO by three NOS isoforms: endothelial NOS (eNOS), neuronal NOS (nNOS), and inducible NOS (iNOS). These isoforms exhibit distinct expression patterns and regulatory mechanisms. eNOS, expressed in vascular endothelial cells, regulates vascular tone and blood pressure via NO-mediated activation of soluble guanylate cyclase (sGC), leading to increased cyclic guanosine monophosphate (cGMP) and subsequent vasodilation via protein kinase G-mediated signaling. nNOS and eNOS isoforms are Ca^2+^/calmodulin-dependent enzymes, whereas the iNOS operates in a Ca^2^-independent manner, likely because calmodulin remains tightly bound to the enzyme even at basal Ca^2+^ concentrations [21]. nNOS is expressed in nervous tissue and skeletal and cardiac muscle, and is involved in synaptic plasticity, neurovascular coupling, and regulation of contractility and mitochondrial biogenesis [22]. iNOS is typically upregulated during inflammation, producing high and sustained levels of NO as part of the immune response [1]. Dysregulation in this pathway, such as BH_4_ deficiency or oxidative uncoupling of NOS, can result in the production of primary reactive oxygen species (ROS) such as superoxide anion (O_2_^−^) rather than NO, contributing to vascular oxidative stress. NO, synthesised through this pathway, also inhibits platelet aggregation, modulates leukocyte adhesion, and exerts antioxidant effects, thus contributing to vascular integrity and cardioprotection [[23], [24], [25], [26]].

Exercise-induced NO production plays a central role in cardiovascular adaptations, including improved endothelial function, angiogenesis, and vascular remodelling [27]. Animal studies have shown that endurance training upregulates NOS expression, particularly eNOS in vascular tissues [28] and nNOS in skeletal muscle [29,30]. In humans, several studies [[31], [32], [33]], but not all [32], have reported increases in saliva and plasma NO_3_^−^ and NO_2_^−^ concentrations following periods of endurance exercise training. A recent systematic review and meta-analysis of studies using chemiluminescence, a reliable technique for measuring NO_3_^−^ and NO_2_^−^ in biological samples, showed higher circulatory levels of NO_3_^−^ and NO_2_^−^ in trained individuals compared to healthy active individuals and unhealthy individuals [34]. Although NO_2_^−^ is considered a more sensitive marker of acute changes in eNOS activity [35], it must be emphasised that circulating NO_3_^−^ concentrations are typically one to two orders of magnitude higher than NO_2_^−^ and reflect contributions from multiple sources, including endogenous NO oxidation, dietary intake, and renal handling [2]. Due to its chemical stability, NO_3_^−^ does not readily undergo direct reduction to NO_2_^−^ or NO in the systemic circulation and therefore should not be interpreted as a sensitive marker of acute eNOS-derived NO production. In contrast, NO_2_^−^ is more dynamically regulated and more closely associated with short-term changes in endothelial NO synthase activity and tissue redox status. Accordingly, while NO_3_^−^ constitutes a large and relatively stable reservoir of nitrogen oxides, its conversion to bioactive NO is highly context-dependent and relies predominantly on enterosalivary cycling or hypoxic and acidic microenvironments. In addition to vascular effects, exercise training enhances NO synthesis within skeletal muscle by upregulating nNOS [36].

## 3 The NOS pathway feeds the nitrate-nitrite-nitric oxide pathway

Historically, NO_3_^−^ and NO_2_^−^ were viewed as inert products of NO oxidation or as undesirable substances found in food and water [37]. However, this view has substantially changed over the last two decades [38]. NO synthesised via the NOS pathway is rapidly oxidised to NO_3_^−^ and NO_2_^−^ through reactions with oxygen, oxyhemoglobin and oxymyoglobin, or reactive oxygen species (ROS). Approximately 25 % of circulatory NO_3_^−^ from endogenous (NO synthesis) or exogenous (diet) sources is actively taken up from plasma to the salivary glands via the sialin transporter (encoded by SLC17A5) and secreted into saliva, resulting in higher NO_3_^−^ levels in saliva than in plasma [39]. Then, commensal anaerobic bacteria equipped with NO_3_^−^ reductase enzymes partially convert NO_3_^−^ into NO_2_^−^ [40]. Alternatively, NO_2_^−^ may be absorbed into the systemic circulation and stored in red blood cells (RBC), which contain the majority of intravascular NO_2_^−^ in whole blood [41,42]. In addition, when elevated concentrations of NO_2_^−^ are ingested, rapid protonation occurs within the acidic environment of the stomach, generating nitrous acid, which subsequently decomposes into NO and other bioactive nitrogen oxides, including HNO_2_, N_2_O_3_, and NO_2_ [43,44]. These species act as nitrosonium (NO^+^) carriers capable of reacting chemically with thiol groups of proteins to form reactive S-nitrosothiols (RSNO), as first identified by Jonathan Stamler in 1992 [45]. This mechanism of NO_3_^−^ bioactivation into NO_2_^−^ and other NO-related species involves a cascade of complex biochemical transformations that are highly dependent on the gastric acidic milieu and are markedly attenuated by drugs that elevate gastric pH [[46], [47], [48], [49]]. Thus, the stomach may be considered a bioreactor that converts dietary NO_2_^−^, or NO_3_^−^-derived NO_2_^−^, into bioactive NO species. Notably, the formation of RSNO, potent NO carriers, represents a central step in this pathway and may also drive important post-translational modifications in protein targets, including pharmacological receptors, through nitrosation—particularly of cysteine residues [24,50]. Beyond these nitrosative mechanisms affecting mediators of blood pressure regulation, NO_2_^−^ can also be absorbed into the circulation and systemically reduced to NO within the RBC by enzymes or proteins with NO_2_^−^-reductase activity, such as XOR or deoxyhemoglobin [51]. Under these conditions, NO generation occurs predominantly during hypoxia, when oxygen availability is limited for eNOS- and nNOS-mediated NO synthesis [41,52]. This mechanism may be particularly relevant during intense physical exercise, where it may help maintain oxygen delivery to hypoxic regions via deoxyhemoglobin-mediated NO generation. Additionally, the inter-connection between these pathways has been linked to some of the cardiovascular benefits of exercise, including post-exercise hypotension, a physiological phenomenon characterised by a sustained reduction in arterial blood pressure following a bout of exercise [[53], [54], [55]].

Importantly, while the l-arginine–NOS pathway is oxygen-dependent, the NO_3_^−^-NO_2_^-^- NO pathway becomes increasingly active as oxygen tensions fall [22,56]. These two pathways are therefore considered complementary, with the NO_3_^−^-NO_2_^-^-NO pathway functioning as a compensatory mechanism to sustain NO production under hypoxic conditions, analogous to how glycolysis supports adenosine triphosphate (ATP) turnover when oxygen is limited [57]. Despite the physiological relevance of this alternative pathway, especially in the context of exercise, no studies to date have examined the effects of exercise training on NO_2_^−^ levels and NO synthesis within RBC or other tissues. Skeletal muscle NO_2_^−^ (and NO_3_^−^) could be measured before and after training, but this does not directly reflect NO synthesis. The latter could, in principle, be quantified pre- and post-training using stable isotopes combined with arteriovenous balance sampling; however, such an approach would not distinguish between NO formation occurring within myocytes and that within RBC. This represents a critical gap in the literature that warrants further investigation to elucidate the role of physical activity in modulating NO metabolism.

**Fig. 1:**
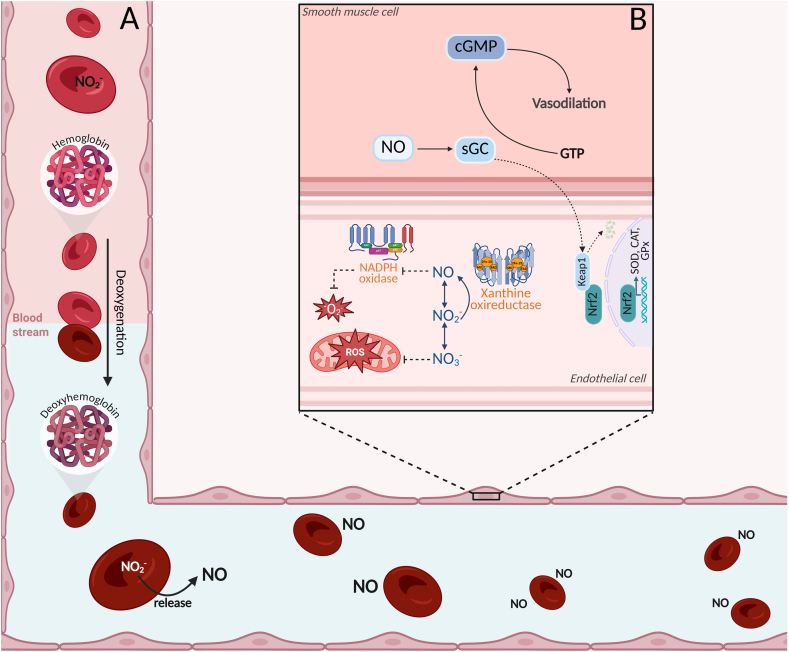
Nitrite reduction in red blood cells and its integration with NOS pathways under low oxygen tension. *Figure created using BioRender.* Legend: The schematic depicts the uptake of circulating NO_2_^−^ into red blood cells and its reduction to bioactive NO by nitrite-reductase systems, including deoxyhemoglobin and, where relevant, xanthine oxidoreductase. This oxygen-independent NO-generating pathway becomes increasingly active when oxygen availability is limited for NOS-dependent NO synthesis, such as during intense physical exercise (Part A). The integration of nitrite-derived NO production with endothelial nitric oxide synthase (NOS) pathways contributes to the maintenance of blood flow in hypoxic tissues and is implicated in exercise-induced cardiovascular adaptations, including post-exercise hypotension (Part B). GTP: Guanosine triphosphate; cGMP: Cyclic guanosine monophosphate; sGC: soluble guanylate cyclase; NADPH: Nicotinamide adenine dinucleotide phosphate (reduced form); ROS: Reactive oxygen species; Nrf2: Nuclear factor erythroid 2–related factor 2; SOD: Superoxide dismutase: CAT: Catalase: GPx: Glutathione peroxidase.

In addition to NO_2_^−^ storage and reduction, RBC have been shown to express an active and functional eNOS isoform localized in the plasma membrane and cytoplasm [42]. Under normoxic conditions, the eNOS-NO pathway is likely the predominant NO-derived pathway in RBC, whereas under hypoxia, NO_2_^−^ reduction by deoxyhemoglobin becomes the primary source (Fig. 1). Interestingly, acute moderate endurance exercise has been shown to activate eNOS in RBC via the PI3K/Akt signalling pathway, which can potentially serve as another source of NO_2_^−^ for RBC [58]. However, the effects of chronic exercise interventions on these and NO_2_^−^-deoxyhemoglobin pathways remain poorly understood and merit further exploration.

**Fig. 2:**
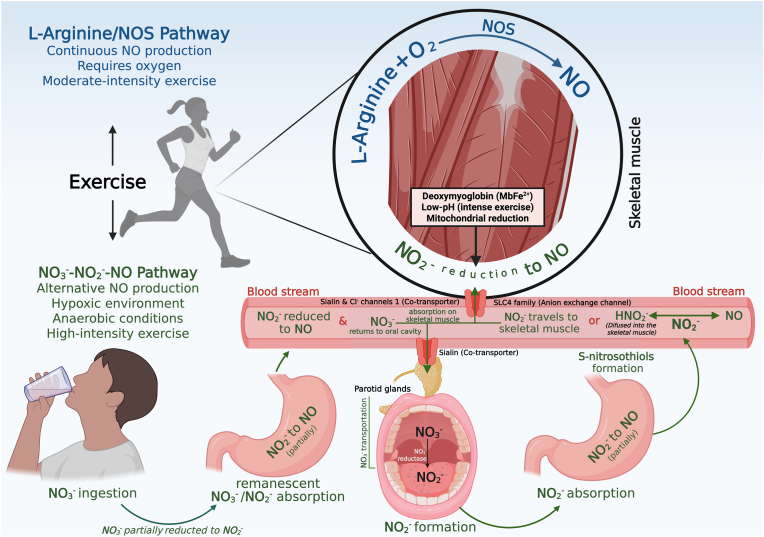
Integration of nitric oxide production pathways in skeletal muscle during physical exercise**.***Figure created using BioRender.* Legend: This figure demonstrates two distinct pathways for nitric oxide production integrated within skeletal muscle. Upper area (oxygen): l-arginine/NOS pathway; Lower area (oxygen-independent): NO_3_–NO_2_^-^-NO path. Under aerobic conditions, nitric oxide is continuously produced via the oxidation of l-arginine by nitric oxide synthase. Conversely, during anaerobic or hypoxic exercise conditions, nitric oxide is synthesised through the nitrate-nitrite-nitric oxide pathway, relying on oral microbiota to reduce at least a portion of nitrate to nitrite, which subsequently forms nitric oxide independently of oxygen availability.

Skeletal muscle expresses all three NOS isoforms (nNOS, iNOS and eNOS), with nNOS being the predominant contributor to NO production in this tissue [59]. Notably, the nNOS isoform in skeletal muscle differs slightly from its neuronal counterpart in other tissues, such as the brain, due to the presence of an additional 34-amino acid segment that includes potential phosphorylation sites [60]. Within the skeletal muscle, nNOS is primarily localised to the sarcolemma of fast and slow-twitch fibres and the neuromuscular junction [61,62]. NO produced by nNOS plays a key role in improving blood flow to the active muscle, modulating mitochondrial respiration and muscle force production, as well as regulating glucose uptake [[63], [64], [65]]. Endurance exercise training has been shown to upregulate nNOS expression in both animal models [30,66] and humans [67], potentially enhancing NO synthesis and increasing levels of its oxidation products, NO_3_^−^ and NO_2_^−^. Whilst emerging evidence suggests that the skeletal muscle serves as an active reservoir for NO_3_^−^ and NO_2_^−^, it has also been suggested that NO_2_^−^ can be reduced to NO under specific physiological circumstances [18]. This reduction can occur via deoxymyoglobin-mediated pathways, low-pH–dependent chemical reactions that become prominent during intense exercise, and mitochondrial nitrite reduction involving components of the electron transport chain, such as cytochrome *c* oxidase. Additionally, skeletal muscle is capable of absorbing circulating NO_3_^−^ through the expression of the sialin and chloride channel 1 [68], which act as transporters to facilitate NO_3_^−^ uptake across cell membranes [69]. Fig. 2 illustrates the integrative interaction between exercise-stimulated nitric oxide synthase (NOS) activity and the NO_3_^−^-NO_2_^–^–NO pathway, depicting how enhanced NOS-derived NO production with exercise contributes to sustained NO bioavailability through providing substrate for the complementary NO_3_^−^-NO_2_^–^–NO pathway.

Despite these insights, the impact of exercise training on intramuscular NO_3_^−^ and NO_2_^−^ levels remain unclear. While some studies in rodents [70] and healthy individuals [71] have reported a reduction in muscle NO_2_^−^ without changes in NO_3_^−^ following prolonged training, and other studies conducted in patients with peripheral arterial disease [72] and healthy individuals [69] have found no significant alterations in NO_3_^−^ or NO_2_^−^. These discrepancies highlight the need for further investigation, particularly using a stable isotope tracer approach, to elucidate the dynamics of NO_3_^−^ and NO_2_^−^ metabolism in skeletal muscle and their contribution to exercise-induced adaptations. Furthermore, leveraging the discussion about the interplay of exercise on NO_2_^−^-NO_3_^-^-NO pathway, preliminary evidence has suggested that elevated plasma NO_2_^−^ concentrations (after NO_3_^−^ ingestion) decline after high-intensity exercise, but not at low-intensity exercise [73].

## 4 Exercise training and the oral microbiome

Facultative anaerobic oral bacteria play a key role in the NO_3_^−^-NO_2_^-^-NO pathway by reducing NO_3_^−^ to NO_2_^−^. Bacteria species with NO_3_^−^ reductase enzymes are, therefore, critical for optimising systemic NO availability [74]. Currently, over 50 oral bacterial species have been identified with NO_3_^−^-reducing capabilities [75], with *Rothia* and *Neisseria* among the most effective in enhancing this conversion. Beyond their role in NO metabolism, NO_3_^−^-reducing bacteria also contribute to oral health by metabolising lactate and producing ammonia [76]. This can help to maintain a more alkaline oral environment, which aids NO_2_^−^ formation [77] and reduces the risk of dental caries and periodontal disease [78]. A potential limitation of the NO_3_^−^-NO_2_^-^-NO pathway in humans arises from the competing microbial process of dissimilatory NO_3_^−^ reduction to ammonium (DNRA), through which oral or gut bacteria reduce NO_3_^−^ to ammonium (NH_4_^+^) under low-oxygen, carbon-rich conditions [79,80]. While DNRA conserves nitrogen for microbial growth, it diverts NO_3_^−^ and NO_2_^−^ away from reductive pathways leading to NO formation, thereby constraining systemic NO bioavailability. Recent analyses of oral NO_3_^−^ metabolism suggest that DNRA occurs in oral microbial consortia (competing with denitrification) and may reduce NO yield in the mouth [79,80].

Dietary NO_3_^−^ intake in the form of beetroot juice (BRJ) [[81], [82], [83], [84]], lettuce juice [85], and green leafy vegetables [86] has been shown to alter the composition of the oral microbiome [87], increasing the relative abundance of *Rothia* and *Neisseria* and reducing the abundance of *Prevotella* and *Veillonella species* [88]. Collectively, these microbial shifts are considered indicative of a healthier oral microbiome profile, potentially enhancing the effectiveness of the NO_3_^−^– NO_2_^−^–NO pathway [76,86]. Although the increase in circulating NO_3_^−^ evoked by exercise is modest compared to dietary supplementation, it may still influence the oral microbiome composition [71]. However, the extent to which exercise alone can modulate oral microbial composition remains poorly understood.

Another mechanism by which exercise may modulate microbial populations in the gut and oral cavity is through the systemic elevation of lactate. During high-intensity exercise, the upregulation of glycolysis leads to a substantial increase in lactate production, which is released into the circulation [89]. While lactate has historically been viewed as a metabolic byproduct destined for hepatic gluconeogenesis via the Cori cycle [90], recent findings suggest that a portion of circulating lactate is transported into the gastrointestinal tract, where it serves as a carbon source for specific microbial taxa [91]. Notably, bacteria from the *Veillonella* genus have been shown to metabolise lactate into short-chain fatty acids (SCFAs) [91], which are known to exert beneficial effects on host metabolic health [92].

Interestingly, *Veillonella species* are also present in the oral cavity, and elevated lactate levels have been detected in saliva following intense exercise [93], suggesting that lactate may similarly serve as a substrate for oral microbial metabolism. A recent and pioneering study investigating the effects of an 8 wk high-intensity interval training (HIIT) program in previously untrained individuals reported significant shifts in the oral microbiome, including an increased relative abundance of *Veillonella species* [71]. This genus includes several NO_3_^−^-reducing bacteria, which may contribute to enhanced NO bioavailability [93]. However, the study did not assess functional changes in oral NO_3_^−^-reducing capacity, leaving another important gap in understanding the physiological relevance of these microbial shifts [71].

Further supporting the role of the oral microbiome in exercise physiology, a moderate positive correlation has been reported between the NO_3_^−^-reducing activity of oral bacteria and maximal aerobic capacity ($\dot{V}$O_2_max) in healthy individuals [94]. These findings suggest that exercise training may enhance both the abundance and functional activity of NO_3_^−^-reducing bacteria in the oral cavity, potentially improving NO bioavailability, particularly under hypoxic conditions where the l-arginine–NOS pathway is less active. Similar findings have been recently reported in patients with heart failure with reduced ejection fraction (HFrEF) [95]. In this regard, whether exercise training restores these oral bacterial functions in HFrEF remains to be tested.

An additional mechanism by which exercise training may support oral health and changes in the oral microbiome is through an increase in salivary flow rate [96]. Saliva plays a crucial role in maintaining oral homeostasis by neutralizing acids, clearing food debris, and delivering antimicrobial proteins and enzymes that inhibit bacterial growth [97]. Enhanced salivary flow contributes to a more stable oral environment, reducing the risk of dental caries and periodontal disease [98]. Evidence suggests that regular exercise training can increase salivary flow rate, which is often accompanied by elevated levels of salivary proteins and electrolytes [96]. These changes may enhance the protective functions of saliva, including buffering capacity and antimicrobial activity. This exercise-induced stimulation of salivary secretion may therefore represent an additional, underappreciated pathway through which physical activity contributes to oral and systemic health [96].

In contrast to the potential positive effects of exercise training, certain dietary practices common among athletes may negatively impact oral health and the microbiome [99]. Frequent consumption of high-sugar sport drinks has been associated with lower species richness and higher abundance of acidogenic bacteria such as *Bifidobacteriaceae* and *Lactobacillus rhamnosus* [100]. These microbial changes have been suggested to contribute to the higher prevalence of periodontal disease observed in athletic populations [[101], [102], [103], [104], [105], [106]]. Additionally, a low-carbohydrate and high-fat diet (keto diet) can also alter the oral microbiome in athletes. A recent study found that this type of diet reduced oral bacterial diversity and abundance of genera such as *Veillonella* and *Prevotella,* which contain NO_3_^−^-reducing species [107]. However, the impact of these dietary impacts on NO_3_^−^-reducing bacteria and the NO_3_^−^–NO_2_^-^–NO pathway in athletes remains to be elucidated.

**Fig. 3:**
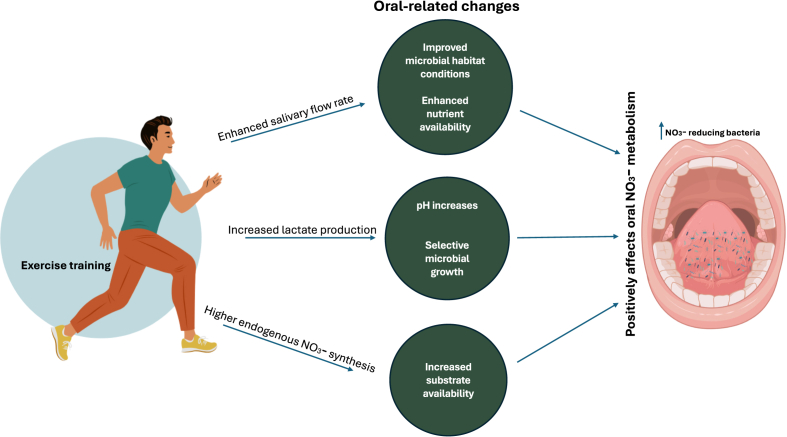
Mechanisms by which physical exercise influences oral microbiome composition. *Figure created partially using BioRender.* Legend: This figure illustrates key mechanisms by which physical exercise influences oral microbiome composition. Exercise enhances endogenous nitrate synthesis, increases lactate production, and modulates oral pH through changes in salivary flow and buffering. These responses vary with exercise intensity, as higher intensities can induce hyperventilation and reduce salivary flow rate, further affecting the oral environment. Collectively, these mechanisms drive shifts in oral microbiota that favor nitrate-reducing bacterial populations, with overall effects influenced by exercise duration and hydration status.

Taken together, these findings suggest that regular physical exercise may promote oral and systemic health not only through direct physiological mechanisms but also via symbiotic microbiome-mediated pathways. However, these microbial changes may be positively or negatively influenced by dietary and oral hygiene habits. Future studies should investigate the functional implications of these shifts, particularly concerning dietary modulation and long-term oral and systemic effects. In summary, exercise-induced increases in endogenous NO synthesis, lactate production, and salivary flow collectively establish a biochemical environment that may promote the proliferation and activity of NO_3_^−^-reducing bacterial taxa, thereby modulating oral NO_3_^−^ metabolism and enhancing downstream NO bioavailability. These interconnected mechanisms are illustrated in Fig. 3 and summarised in detail in Table 1.

**Table 1:** Summary of studies investigating the interaction between exercise training, oral microbiota composition, and nitrate-reducing bacteria (NRB).

Reference	Design & Population	Exercise/Exposure	Oral Microbiota — method, site, timepoints	Changes in NO_3_^−^-reducing bacteria	Nitrate/Nitrite/NO outcomes	Main conclusion
Simpson et al., [71]	n = 11 untrained males; (one-arm) baseline → post-8wk HIIT → 12wk detraining	8-wk HIIT (cycle ergometer), ∼3 × /wk, 16–36 min per session; ∼75 min/wk; 12-wk detraining	PacBio long read 16S rRNA; tongue dorsum; baseline/post/detraining; NO_3_^−^ & NO_2_^−^by HPLC in saliva, plasma, muscle	Genus: ↑ Rothia, ↑ Prevotella; ↓ Neisseria; ↔ Veillonella. Species: ↑ multiple NO_3_^−^-reducing spp., incl. *Rothia mucilaginosa*; *Streptococcus salivarius*.	Post-training: ↑ plasma NO_3_^−^; ↑ salivary NO_2_^−^;↓ plasma NO_2_^−^; ↓ muscle NO_2_^−^. Some NO_3_^−^-reducing spp. remained elevated after detraining.	HIIT altered the tongue microbiome with enrichment of several NO_3_^−^-reducing taxa; bioavailability shifts were observed across compartments.
Simpson et al., [108]	n = 10 highly trained athletes vs n = 10 untrained controls	Cross-sectional (habitual training status; VO_2_max assessed)	PacBio long read 16S rRNA; tongue dorsum & supragingival plaque; saliva & plasma NO_3_^−^/NO_2_^−^	Tongue: Athletes had ↑ *Rothia mucilaginosa* (confirmed NRB) and ↑ Gemella (unclassified spp.). Beta-diversity differed (tongue).	Athletes: ↑ salivary NO_3_^−^ and ↑ salivary NO_2_^−^.	Habitual training is associated with a higher abundance of specific NO_3_-reducing bacteria (esp. *R. mucilaginosa*) and higher salivary NO_3_^−^/NO_2_^−^.
Urban et al., [109]	n = 20 professional male footballers vs n = 12 amateurs	Cross-sectional; periods of intensive vs non-intensive training analysed	16S rRNA sequencing; oral & fecal microbiota (oral site: saliva/combined oral swab)	Oral after intensive activity: ↑ Neisseriaceae (incl. *Neisseria perflava*); ↑ Prevotellaceae; ↑ *Rothia dentocariosa* and ↑ *Rothia aeria*.	Salivary, plasma, or skeletal muscle NO_3_^−^/NO^2−^ values not reported.	Intensive activity associated with shifts in oral taxa, including NO_3_-reducing bacteria genera (Neisseria, Rothia).
Stahl et al. [95]	n = 9 patients with HFrEF vs n = 9 healthy controls	Cross-sectional: (Oral NO_3_^−^-reducing capacity between patients with HFrEF vs healthy adults)	Not assessed	Not assessed	Oral NO_3_^−^-reducing capacity and saliva NO_2_^−^ concentrations: ↓ in HFrEF	Oral NO_3_^−^-reducing capacity is impaired in patients with HFrEF compared to age-matched healthy adults

*Legend:* wk: Week; NaCl: sodium chloride; NRB: Nitrate-Reducing Bacteria; NO: Nitric Oxide; NO_3_^−^: Nitrate; NO_2_^−^: Nitrite; HFrEF: Heart Failure with Reduced Ejection Fraction; HIIT: High-Intensity Interval Training; HPLC: High-Performance Liquid Chromatography; RCT: Randomized Controlled Trial; VO_2_max: Maximal Oxygen Uptake (aerobic fitness measure); 16S rRNA: 16S ribosomal RNA gene sequencing (microbiota profiling). Symbols denote direction of change: ↑ increase, ↓ decrease, ↔ no change. These directions are illustrated based on statistical inference in the original papers, with significance attributed to *p* < 0.05.

The information illustrated in Table 1 highlights information from studies involving exercise training or physical performance outcomes related to NO oral metabolism.

## 5 Dietary inorganic NO_3_^−^ effects on exercise outcomes

Dietary NO_3_^−^ is naturally present in food [3,110], especially in green leafy vegetables and beetroot [[111], [112], [113], [114], [115], [116], [117], [118], [119]]. Consumption of NO_3_^−^−rich foods can significantly elevate circulating NO_3_^−^ levels for several hours, with the duration and magnitude of this increase influenced by dose and individual health status [[120], [121], [122]]. For example, the half-life of circulating NO_3_^−^ is prolonged in individuals with impaired renal function [123]. Over the past two decades, a substantial number of studies have investigated the potential ergogenic effect of dietary NO_3_^−^ supplementation, with almost all NO_3_^−^ studies using doses varying from 6.4 mmol to 12.8 mmol (∼400 mg–800 mg of NO_3_^−^). Overall, dietary NO_3_^−^ appears to be more effective in evoking performance improvements in moderately active individuals than in endurance-trained athletes [[124], [125], [126], [127], [128], [129], [130], [131]]. This may be attributed to the superior aerobic capacity of endurance-trained individuals, in whom upregulation of eNOS and nNOS, enhanced antioxidant defences, and improved NOS coupling collectively augment NOS-derived NO bioavailability [36,58,132]. In addition, their predominance of slow-twitch muscle fibres [133,134], which are less prone to hypoxia and acidosis during exercise, further diminishes reliance on exogenous NO precursors, particularly under conditions where aerobic metabolism and NOS-derived pathways predominate. Supporting this, Porcelli et al. [135] found a negative association between aerobic fitness levels ($\dot{V}$O_2max_) and plasma NO_3_^−^ and NO_2_^−^ levels following NO_3_^−^ supplementation. Another important factor influencing the efficacy of NO_3_^−^ supplementation is individual variability in the conversion of NO_3_^−^ to NO_2_^−^. Christensen et al. [136] found that athletes with high baseline plasma NO_2_^−^ levels and a lower capacity to convert NO_3_^−^ to NO_2_^−^ experienced reduced performance benefits from supplementation. Coggan et al. [137] demonstrated a modest but significant correlation between plasma NO_2_^−^ increases following NO_3_^−^ intake and improvements in muscle contractile function. Similarly, Hoon et al. [138] reported a moderate positive association between changes in plasma NO_2_^−^ and performance improvements in well-trained rowers after acute ingestion of 8.4 mmol of NO_3_^−^ in the form of BRJ.

As previously discussed, the oral microbiome plays a key role in the reduction of NO_3_^−^ to NO_2_^−^. Differences in oral microbial composition and activity may, therefore, contribute to the variability in NO_3_^−^ metabolism in response to dietary NO_3_^−^ ingestion. A recent study by Simpson et al. [71] has shown that HIIT during 8 wk increased the abundance of NO_3_^−^-reducing bacteria in non-trained individuals. Furthermore, a recent pilot study showed differences in oral microbiome composition between highly trained competitive athletes to untrained controls, demonstrating that highly trained individuals may have a higher ability to produce NO through the NO_3_^−^-NO_2_^-^-NO pathway [108]. Future research should investigate whether elite athletes exhibit higher activity of NO_3_^−^-reducing bacteria and whether these levels fluctuate across different phases of the training season.

In pathologies such as chronic heart failure (CHF), endothelial dysfunction and reduced eNOS activity impair NO production via the NOS pathway. In such cases, dietary NO_3_^−^ supplementation may help restore NO bioavailability. In a murine model with CHF [139] 5 d of inorganic NO_3_^−^ supplementation enhanced skeletal muscle blood flow and vascular conductance during submaximal aerobic exercise, alongside reductions in mean arterial pressure and blood lactate. Notably, these improvements were more pronounced in fast-twitch, glycolytic muscles—fibers that experience greater oxygen desaturation and acidosis during exercise, conditions favouring NO_2_^−^ reduction to NO. Similarly, 5 d of NO_3_^−^ supplementation has been shown to improve exercise tolerance in mice with sickle cell disease, a condition characterised by impaired muscle function and reduced NO bioavailability [139]. Evidence from human studies in patients with heart failure indicates mixed effects of NO_3_^−^ supplementation [[140], [141], [142], [143], [144]]. Coggan et al. [141] reported that acute BRJ improved $\dot{V}$O_2_peak and time to fatigue in patients with HFrEF. In contrast, Hirai et al. [140] found no improvement in exercise intolerance following 9 d of BRJ ingestion in a similar cohort. In contrast, Kerley et al. [145] demonstrated gains in exercise tolerance during the incremental simulated walking test in HFrEF patients, whereas Woessner et al. [142], following 5 d of BRJ ingestion, observed no significant changes in $\dot{V}$O_2_peak or time to exhaustion. Collectively, these discrepant findings highlight that the ergogenic and vascular benefits of NO_3_^−^ supplementation in heart failure populations remain inconclusive and reflect heterogeneity in supplementation duration. In this line, a recent large study led by Zamani et al. [144] involving eighty-four patients with heart failure with preserved ejection fraction reported no improvement in exercise capacity with potassium nitrate supplementation over 6 wk, highlighting that responsiveness to NO_3_^−^ may vary by disease subtype and therapeutic context. In other populations, such as patients with peripheral artery disease, studies have reported improved exercise and cardiovascular performance following NO_3_^−^ supplementation in patients with peripheral artery disease [[146], [147], [148]].

One confounding factor is that clinical patients commonly take medications (e.g., antihypertensives, statins, XOR inhibitors, and angiotensin-converting enzyme inhibitors) that can modulate NO metabolism and thus alter their responsiveness to oral NO_3_^−^ supplementation [51,149]. Interestingly, in human forearm studies, NO_2_^−^ infusion can restore the increase in blood flow even when NOS is inhibited, via reduction by deoxyhemoglobin (i.e. NO_2_^−^ to NO) [150]. Even with previously mentioned limitations, a recent systematic review with meta-analysis showed that enhancing the NO_3_^−^–NO_2_^-^–NO pathway using inorganic NO_3_^−^– before exercise had a positive effect to modulate the blood pressure response during and after exercise, especially in patients with high resting blood pressure levels [151]. Increased flow-mediated dilation, assessed in some of these studies, reveals that NO_3_^−^ can offset some of the cardiovascular strain independently of the effectiveness of eNOS activity [121,152].

Aging is also associated with impaired NO bioavailability [153], potentially contributing to declines in vascular and muscular function. Dietary NO_3_^−^ interventions have been proposed as a strategy to mitigate these effects in older adults [6,[154], [155], [156], [157]]. Supporting this, a preliminary randomised controlled trial by Benjamim et al. [158] demonstrated that 8 d of BRJ (NO_3_^−^ 6.4 mmol/day) improved the 6 min walking test in postmenopausal women. In line with these findings, several studies, but not all [159], have reported improvements in muscle function and exercise tolerance following NO_3_^−^ supplementation in older individuals [[160], [161], [162], [163]]. Another recent randomised trial led by Alvares et al. [164] demonstrated that 12 wk of NO_3_^−^-rich beetroot extract (8.8 mmol) supplementation significantly enhanced femoral artery endothelial function, tibialis anterior muscle microvascular reactivity, and serum angiogenic potential in postmenopausal women, highlighting NO_3_^−^ as a promising dietary strategy to counteract vascular decline and reduce cardiovascular risk in this population [164].

## 6 Combination of exogenous (**NO_3_^−^** supplementation) and endogenous (exercise training) stimulation of **NO_3_^−^** and **NO_2_^−^** bioavailability in exercise physiology

The aspects previously mentioned suggest that exercise combined with oral NO_3_^−^ administration can lead to synergistic effects on performance or health outcomes. Although this area needs further exploration, a few preliminary studies have been published on this topic in the past few years. A recent randomised study has demonstrated that chronic dietary NO_3_^−^ intervention can enhance the benefits of exercise training in late postmenopausal women. In Carter et al.’s study [165], participants engaged in a structured program of circuit-based exercise for 8 wk, and those who combined their workouts with pre-exercise BRJ (NO_3_^−^ 12.8 mmol) supplementation exhibited additional benefits. Indeed, compared with exercise alone, the NO_3_^−^-supplemented group experienced greater improvements in the 6 min walking test, cardiorespiratory fitness ($\dot{V}$O_2_peak), and recovery of heart rate after exercise—key indicators of functional capacity and cardiovascular health in older women. The findings also suggested potential benefits for muscle power and exercise efficiency, though these trends were less pronounced [165]. The results of this longer-term combined intervention contrast with shorter studies involving older adults, such as Siervo et al. [166], who, after 1 wk of BRJ (NO_3_^−^ 12.8 mmol) ingestion alone, did not observe significant benefits to exercise performance in older male and female adults.

The combination of dietary NO_3_^−^ with other NO precursors has also been explored to enhance NO bioavailability and physiological outcomes. Le Roux-Mallouf et al. [167] investigated the effects of 8 wk of combined endurance and resistance training alongside daily supplementation of NO_3_^−^ (∼8.4 mmol NO_3_^−^·day^−1^) and citrulline (6 g·day^−1^) in healthy older adults. The intervention led to greater improvements in knee extensor maximal voluntary contraction compared to training alone, although no additional benefits were observed in aerobic performance or vascular function. Notably, the absence of a group receiving only NO metabolites (e.g., NO_3_^−^) and a separate non-exercising control group in this study limited the ability to determine whether the combined supplementation would be more effective than either precursor alone. Studies using l-citruline supplementation alone have shown inconsistent results regarding its ability to increase NO metabolites (e.g., NO_3_^−^ and NO_2_^−^) [168,169]. Similarly, the combination of NO_3_^−^ and l-arginine supplementation did not increase plasma NO metabolites nor improve exercise performance in elite athletes [170], suggesting limited synergistic potential between these precursors under certain physiological conditions [166,167].

Other studies have focused on the interaction between exercise training and NO_3_^−^ supplementation, particularly using NO_3_^−^-rich BRJ, to enhance NO bioavailability and physiological effects. Following this approach, Thompson et al. [171] demonstrated that 4 wk of sprint interval training (SIT) combined with BRJ supplementation (∼13 mmol NO_3_^−^·day^−1^) in healthy active individuals improved submaximal exercise efficiency, evidenced by reduced oxygen consumption ($\dot{V}$O_2_) at moderate intensity. Additionally, metabolic responses were altered, including lower blood lactate and higher muscle pH, although performance at severe intensity remained unchanged. Notably, plasma NO_3_^−^ levels were higher in the combined SIT and supplementation group compared to supplementation alone, suggesting a synergistic effect of exercise in enhancing NO bioavailability.

Collectively, these results suggest that the combination of NO_3_^−^ supplementation and exercise training may exert synergistic effects on physiological responses. The observed benefits of NO_3_^−^ supplementation may be context-dependent. In healthy individuals undergoing high-intensity training, NO_3_^−^ may support NOS-driven adaptations by providing NO under transient hypoxic and acidic intramuscular conditions. Conversely, in aging or disease states characterised by impaired NOS activity, dietary NO_3_^−^ may serve as a primary NO source via the NO_3_^−^-NO_2_^-^-NO pathway, restoring vascular control and oxygen delivery. Future research should investigate the interplay between these pathways, the influence of oral microbiota composition on NO_3_^−^ reduction capacity, and the dose–response relationship in both athletic and clinical populations.

**Fig. 4:**
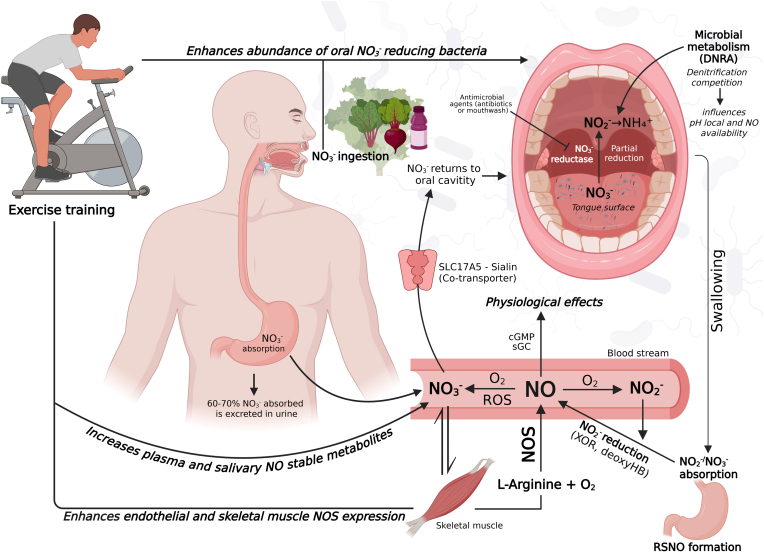
This figure demonstrates the integration of nitric oxide synthesised by exercise training and how it can feed the NO_3_^−^–NO_2_^-^–NO pathway (via inorganic nitrate ingestion). *Figure created using BioRender.* Legend: Nitric oxide is synthesised by nitric oxide synthase enzymes from the l-Arginine pathway and reduced to nitrate in systemic circulation. Nitrate returns to the oral cavity (saliva) using a co-transporter (sialin) and is partially reduced to nitrite by nitrate-reducing bacteria (oral microbiota). Nitrite is subsequently absorbed and converted back to bioactive nitric oxide by xanthine reductase (XOR) and deoxyhemoglobin (deoxyHb)or in the stomach, leading to NO derivatives (e.g., S-nitrosothiols) formation. Additionally, some of the reduced nitrate to nitrite is further reduced to ammonium (DNRA pathway), diverting substrates away from the nitrate–nitrite-nitric oxide pathway. Ultimately, endogenous and exogenous pathways contribute to nitric oxide production and its role in activating second messengers, which in turn lead to physiological effects. Nitric oxide is primarily oxidised to nitrite and nitrate in oxygen-rich environments such as arterial blood, where oxygen, oxyhemoglobin, oxymyoglobin, and reactive oxygen species (ROS) are abundant [43]. These ROS-driven reactions are not strictly required for NO oxidation but can markedly enhance NO loss under oxidative stress, linking redox imbalance to reduced NO bioavailability. Circulating NO_3_^−^ is actively transported to the salivary glands via the sialin transporter and reduced to NO_2_^−^ by oral nitrate-reducing bacteria. Swallowed NO_2_^−^ can be protonated in the stomach to form nitrous acid (HNO_2_), which decomposes into bioactive NO and related nitrogen oxides (e.g., N_2_O_3_, NO_2_•, and S-nitrosothiols). In peripheral tissues and blood, NO_2_^−^ is further reduced to NO under low-oxygen conditions by deoxyhemoglobin, xanthine oxidoreductase (XOR), or deoxymyoglobin.

A summary of the integration between exercise-derived nitric oxide synthesis and the NO_3_^−^-NO_2_^-^-NO pathway is illustrated in Fig. 4, showing how endogenous NOS activity and dietary NO_3_^−^ ingestion converge to sustain NO bioavailability. Exercise-induced NO is oxidised to NO_3_^−^, recycled through saliva via sialin, and partially reduced to NO_2_^−^- by oral bacteria, which can then regenerate bioactive NO through enzymatic or acidic reduction. A competing pathway, DNRA, may divert substrates away from NO production, limiting overall NO availability.

## 7 Perspectives

It is recognized that NO is a key signalling molecule in human physiology, mediating vascular tone, immune defense, mitochondrial efficiency, skeletal muscle function and various other processes [172]. While historically NO synthesis was derived exclusively through the NOS pathway, the alternative NO_3_^−^– NO_2_^−^–NO pathway is now a recognized target in interventional studies. Within this pathway, the oral microbiota plays a pivotal role in initiating NO_3_^−^ reduction and sustaining systemic NO bioavailability [71]. Notably, exercise training may further modulate this microbiota–NO_3_^-^ interaction by increasing salivary flow, altering oral pH, and improving mucosal blood flow, factors that potentially favor the proliferation and activity of NO_3_^−^-reducing bacteria. Therefore, the integration of NO_3_^−^- and vegetable-rich diets alongside structured exercise training may act synergistically to optimize NO metabolism, not only by stimulating NOS-derived NO production but by enhancing the microbiota-mediated oral NO_3_^−^-NO_2_^-^-NO axis [173].

Although animal studies provide valuable insights into NO_3_^−^ metabolism, profound interspecies differences limit their translational applicability. In humans, dietary NO_3_^−^ is actively concentrated in saliva, where it is reduced by oral bacteria to NO_2_^−^ and subsequently reduced to NO and related species. In contrast, rodents lack this active salivary transport mechanism via sialin, and their salivary NO_3_^−^ and NO_2_^−^ concentrations remain at plasma levels after NO_3_^−^ treatment [13]. Therefore, while preclinical models can aid mechanistic understanding, caution is warranted when extrapolating rodent data to human physiology. Further mechanistic studies directly conducted in humans remain essential, as discussed in the previous sections.

Importantly, beyond NO_3_^−^ and NO_2_^−^ metabolism, oral bacteria produce a wide array of secondary metabolites, such as short-chain fatty acids, reactive oxygen species modulators, and signaling peptides, that may influence host redox status, inflammatory responses, and mucosal immunity. Although these effects are not yet fully characterised in the context of NO metabolism, their contribution to the broader redox landscape should not be overlooked. Future studies should investigate how microbial-derived metabolites influence NO_3_^−^ metabolism and whether they potentiate or buffer NO-related signalling in target tissues. Furthermore, there is a need to study the chronic synergistic effects of both NO_3_^−^-rich diets/supplementation and different exercise training programs. In addition, current evidence largely derives from short-term interventions; the long-term effects of habitual or low-intensity physical activity (e.g., brisk walking) on NO metabolism remain insufficiently characterised and warrant further investigation.

## CRediT authorship contribution statement

**Jonas Benjamim:** Conceptualization, Investigation, Methodology, Project administration, Resources, Validation, Visualization, Writing – original draft, Writing – review & editing. **Stephen J. Bailey:** Writing – original draft, Writing – review & editing. **Leonardo da Silva Gonçalves:** Writing – review & editing. **Mia Burleigh:** Writing – review & editing. **Mario Siervo:** Writing – original draft, Writing – review & editing. **Andrew R. Coggan:** Validation, Writing – original draft, Writing – review & editing. **Raúl Bescos:** Conceptualization, Investigation, Methodology, Project administration, Resources, Supervision, Validation, Visualization, Writing – original draft, Writing – review & editing.

## Declaration of competing interest

The authors declare that they have no known competing financial interests or personal relationships that could have appeared to influence the work reported in this paper.

## Data Availability

No data was used for the research described in the article.
